# Decreased gray matter volume and increased white matter volume in patients with neovascular age-related macular degeneration: a voxel-based morphometry study

**DOI:** 10.18632/aging.203610

**Published:** 2021-10-06

**Authors:** Yan-Kun Shen, Qian-Min Ge, Yi-Cong Pan, Hui-Ye Shu, Li-Juan Zhang, Qiu-Yu Li, Rong-Bin Liang, Yi Shao, Yao Yu

**Affiliations:** 1 Department of Endocrinology and Ophthalmology, The First Affiliated Hospital of Nanchang University, Jiangxi Center of National Ocular Disease Clinical Research Center, Nanchang 330006, Jiangxi, People’s Republic of China

**Keywords:** neovascular age-related macular degeneration, choroidal neovascularization, mental disorder

## Abstract

Objective: To measure white and gray matter volume (WMV, GMV) in patients with neovascular age-related macular degeneration (nAMD) using voxel-based morphometry (VBM).

Material: Eighteen patients (9 men, 9 women) with nAMD and 18 (9 men, 9 women) healthy controls (HCs) aligned were recruited. Functional magnetic resonance imaging (fMRI) and VBM of three-dimensional T1 brain images were analyzed. And we also apply t-tests to look for GMV and WMV differences between groups. Correlation analysis was utilized to probe the connection between observational GMV and WMV values of diverse brain areas and the severity of HADS (hospital anxiety and depression scale). Also, distinctions between nAMD and HCs in GMV can be presented with the help of a ROC (receiver operating characteristic) curve.

Results: Compared with HCs, GMV values were significantly lower in patients with neovascular age-related macular degeneration in the right inferior frontal gyrus, temporal pole of left superior temporal gyrus, left superior temporal gyrus, left middle frontal gyrus, left anterior cingulate and para cingulate gyrus. While WMV was slightly increased in these areas. HADS (hospital anxiety and depression scale) scores portrayed a non-linear correlation with the GMV value of the right inferior frontal gyrus, left middle frontal gyrus, left anterior cingulate and paracingulate gyrus of the nAMD group (r=-0.6629, P=0.0027)(r=-0.9451, P<0.0001)(r=-0.6183, P=0.0062). Moreover, the ROC curve analysis of the mean VBM values for altered brain regions indicated high diagnostic accuracy.

Conclusion: These results indicated that patients with nAMD have abnormal GMV and WMV and formed a basis for future research on pathological mechanisms in this disease. Moreover, decreased gray matter volume in particular brain regions might be associated with choroidal neovascularization and abnormal HADS score. It might help to explain the pathological mechanism of anxiety and depression in patients with nAMD.

## INTRODUCTION

Age-related macular degeneration (AMD) is the third most common potentially blinding eye disease globally, with a significant impact on elderly patients’ quality of life [1]. Its disease stage may be categorized as early, late indeterminate, late wet active, late dry, or late wet inactive. AMD arises and progresses with age, damaging the central (macular) region of the retina, and features no clear pathogeny [2]. As a common cause of severe vision loss, nAMD is accompanied by choroidal neovascularization, an important pathological feature of this disease stage. And a lack of timely intervention may lead to visual impairment and even blindness [3].

The diagnosis of nAMD is mainly based on fundus angiography, optical coherence tomography, and confocal laser fundus imaging [4, 5]. Diversified diagnostic methods and standards being helpful to diagnose nAMD in its different presentations. In addition to medical diagnosis, patients with the following risk factors should be alert to the possible occurrence of nAMD: (1) the elderly; (2) nAMD in the other eye; (3) family history of nAMD; (4) smoking and smoking history; (5) hypertension; (6) obesity or hyperlipidemia; (7) insufficient intake of vitamins, carotenoids. and minerals; (8) high-fat diet; (9) lack of exercise.

With the characteristics of noninvasive, clear imaging and high soft-tissue resolution ratio, magnetic resonance imaging (MRI) has been increasingly applied in the research fields of ophthalmic diseases [6], related animal models [7], and the clinical research of visual injury [8]. Thus, MRI can be used to detect brain changes in nAMD patients and compare all images in the same stereotactic space [9].

Voxel-based morphometry (VBM) is a statistical analysis tool to measure particle size of gray matter [10]. It can be used to measure and compare the volume as well as gyration between groups. Correlation analysis of clinical scores shows that VBM can be used not only for structural analysis but also for analyzing the impact of structural changes [11]. It has been used to study the pathology of trigeminal neuralgia, senile dementia, mild cognitive impairment, and motor neuron diseases [12–14]. In ophthalmology, it has also been used to increase understanding of optic neuritis, retinal detachment, acute eye pain, advanced monocular blindness [15–18], and nAMD. It offers a powerful method of elucidating the pathological mechanisms of those diseases, and monitoring the course of their progression.

## RESULTS

### Demographics and visual surveys

No significant difference in age (P=0.802) or handedness (P>0.99) was found between the nAMD and HC groups. Handedness was analyzed using the chi-squared test. Significant differences were found in the best-corrected right and left visual acuities (P=0.003 and P=0.001 respectively) but not in right or left intraocular pressure (P=0.119 and P=0.134 respectively). Details are shown in Table 1.

**Table 1:** Demographics and behavioral results of nAMD and HCs groups.

	**nAMD**	**HC**	**t-value**	***p*-value**
Male/female	9/9	9/9	N/A	>0.99
Age(years)	55.74±5.32	56.24±5.43	0.311	0.802
Handedness	18R	18 R	N/A	>0.99
Duration (M)	1.16±0.41	N/A	N/A	N/A
Best-corrected VA-L	0.10±0.05	1.05±0.15	7.344	0.001
Best-corrected VA-R	0.15±0.05	0.95±0.15	5.433	0.003
IOP-L	14.59±6.61	14.26±4.22	1.657	0.134
IOP-R	13.63±5.27	13.17±4.26	1.764	0.119

Notes: Independent t-tests comparing the age of two groups (P<0.05) represented statistically significant differences). Male/female and Handedness were analyzed using chi-squared test.

Abbreviations: nAMD, neovascular age-related macular degeneration; HCs, healthy controls; N/A, not applicable; VA, visual acuity; R, right; L, left; IOP, intraocular pressure.

### VBM differences

**Figure 1:**
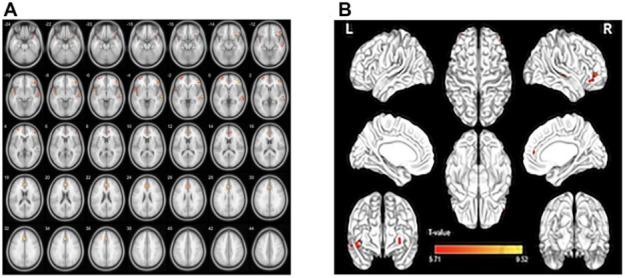
**GMV regional decrease in patients with nAMD compared with HCs.** (**A**, **B**). Notes: Compared with the HC group, the GMV was reduced in the right inferior frontal gyrus in the patients with nAMD, as well as left superior temporal gyrus temporal pole, left superior temporal gyrus, left middle frontal gyrus, left anterior cingulate and paracingulate gyrus. Abbreviations: nAMD, neovascular age-related macular degeneration; HC, healthy controls; GMV, gray matter volume.

Compared with HCs, the GMV values were generally lower in patients with nAMD in the left superior temporal gyrus, right inferior frontal gyrus, left middle frontal gyrus, temporal pole of left superior temporal gyrus, and the left anterior cingulate and para cingulate gyrus (P=0.010). While group differences in WMV were not found within regions, the averaged white matter volume (WMV) was slightly higher in the nAMD group. (Figures 1 [red], 2 and Tables 2, 3; P<0.01 for multiple comparisons by GRF theory).

**Figure 2:**
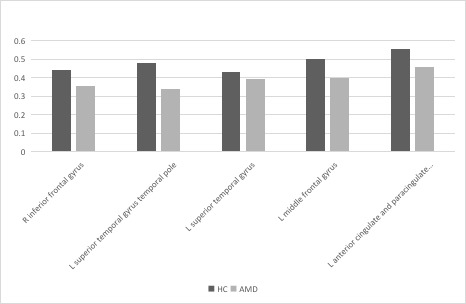
**The mean GMV values between the nAMD and HC group.** Abbreviations: GMV, gray matter volume; nAMD, neovascular age-related macular degeneration; HC, healthy controls.

**Table 2:** Brain regions with significant differences in grey matter volume between nAMD group and HCs.

**Brain areas**	**MNI coordinates**			**Number of voxels**	**T value**
	**X**	**Y**	**Z**		
HC>nAMD					
right inferior frontal gyrus	37.5	18.5	-21.5	74	6.7939
left superior temporal gyrus temporal pole	-46.5	-5.5	-11.5	184	7.2464
left superior temporal gyrus	63.5	-19.5	-3.5	209	7.9122
right inferior frontal gyrus	43.5	44.5	0.5	233	7.8886
left middle frontal gyrus	-32.5	54.5	0.5	119	6.9893
left anterior cingulate and paracingulate gyrus	-0.5	38.5	20.5	458	9.5194

Abbreviations: nAMD, neovascular age-related macular degeneration; HC, healthy controls.

**Table 3:** Group GMV differences between nAMD group and HC group.

	**nAMD group**	**HC group**	**t**	**P**
GMV	594.11±51.10	645.71±51.42	2.736	0.010
WMV	506.67±58.30	501.79±46.65	-0.248	/

Abbreviations: nAMD, neovascular age-related macular degeneration; HC, healthy controls.

### Correlation analysis

**Figure 3:**
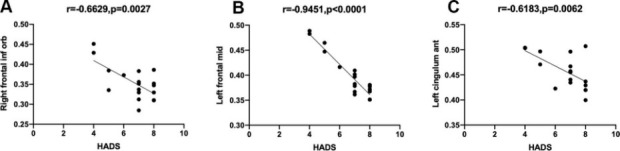
**Correlation between the mean GMV and severity of HADS in different brain areas.** Notes: (**A**) The GMV value of the right inferior frontal gyrus of the nAMD group portrayed a non-linear correlation with the severity of HADS (r=-0.6629, P=0.0027). (**B**) The GMV value of the left middle frontal gyrus of the nAMD group portrayed a non-linear correlation with the severity of HADS(r=-0.9451, p<0.0001). (**C**) The GMV value of the left anterior cingulate and paracingulate gyrus of the nAMD group portrayed a non-linear correlation with the severity of HADS(r=-0.6183, p=0.0062). Abbreviations: GMV, gray matter volume; HADS, hospital anxiety and depression scale; nAMD, neovascular age-related macular degeneration.

In the nAMD group, the GMV values of the right inferior frontal gyrus, left middle frontal gyrus, left anterior cingulate and para cingulate gyrus were each non-linear correlated with HADS scores (r=- 0.6629, P=0.0027; r=-0.9451, P<0.0001; r=-0.6183, =0.0062). The details are shown in Figure 3.

### ROC curve

**Figure 4:**
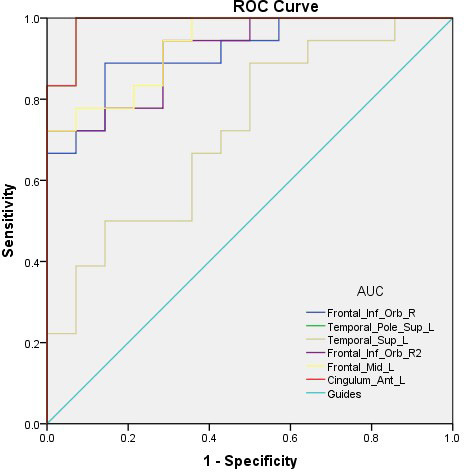
**ROC curve analysis of the mean VBM values for altered brain regions.** Notes: The area under the ROC curve were 0.917 (P<0.0001; 95% CI: 0.823-1.000) for Frontal_Inf_Orb_R, Temporal_Pole_Sup_L 0.988 (P<0.0001; 95% CI: 0.960-1.000), Temporal_Sup_L 0.722 (P=0.033; 95% CI: 0.544-0.900), Frontal_Inf_Orb_R2 0.917 (P<0.0001; 95% CI: 0.824-1.000), Frontal_Mid_L 0.933 (P<0.0001; 95% CI: 0.852-1.000), Cingulum_Ant_L 0.988 (P<0.0001; 95% CI: 0.960-1.000). Abbreviations: AUC, area under the curve; ROC, receiver operating characteristic.

The areas under the curves (AUCs) for GMV values in these regions were as follows: right inferior frontal gyrus=0.917, left superior temporal gyrus temporal pole=0.722, left middle frontal gyrus=0.933, left anterior cingulate and para cingulate gyrus=0.988. Details are shown in Figure 4.

## DISCUSSION

To our knowledge, this is the first study to use the VBM method to investigate differences in GMV and WMV in nAMD patients. The results indicate that the average value of GMV was lower while the average one of WMV was slightly higher in nAMD patients compared with HCs. Within regions, GMV was significantly lower in the right inferior frontal gyrus, left superior temporal gyrus temporal pole, left superior temporal gyrus, left middle frontal gyrus, left anterior cingulate and para cingulate gyrus.

As explained earlier (see Introduction), nAMD is a common macular degeneration disease that progresses slowly and it mainly afflicts the elderly. It is characterized by central vision loss, which often leads to map-like atrophy as well as neovascularization. Choroidal neovascularization is the most essential pathological feature of nAMD and a lack of timely intervention may lead to severe complications, such as visual acuity being reduced by three lines in one year and four lines in two years [19, 20]. Therefore, diagnosis and treatment of the disease need more attention. In past studies, we have used fMRI to assess neural activity in patients with nAMD. While in the present study VBM was used to investigate changes of GMV and WMV in patients with nAMD and to explore the possible underlying pathogenesis.

The results showed reduced GMV in right inferior frontal gyrus in patients with nAMD. The inferior frontal gyrus is located on the ventral side of the inferior frontal sulcus. It is in front of the anterior central sulcus and on the lateral fissure. It is divided into three accessory gyri (Brodmann area 47/12, Brodmann area 45, Brodmann area 44) [21] by the anterior ascending branch and the anterior horizontal branch of the lateral fissure. The most widely known functions of the inferior frontal gyrus are speech and language comprehension [22]. Some results showed that in patients with depression, the resting state amplitude of low frequency fluctuation (ALFF) of the left inferior frontal gyrus was significantly decreased. It was associated with functional abnormalities including emotion, cognition, and memory [23].

The temporal lobe is segmented into superior, middle, and inferior gyri. The superior temporal gyrus is also the vestibular cortex. Therefore, balance disorder and vertigo may arise with temporal lobe lesions. Oculomotor nerve palsy may occur with large lesions of the superior temporal gyrus. With functional magnetic resonance imaging (fMRI), one study showed that patients with left anterior temporal lobectomy behaved activation in the central cover processing structure due to intractable epilepsy. After both right and left lobectomy, the researchers discovered in the right superior temporal sulcus reduced responses to faces together with reduced recognition of facial expression [24]. In addition, damage to the left posterior superior temporal gyrus has been associated with impairments in phonological processing [25]. NAMD may damage the left temporal pole, which may lead to impaired recognition of facial expression, language difficulty, and arachnoid cyst [26]. It can compress brain tissue and cause headache, intractable epilepsy and other corresponding symptoms [27].

The anterior cingulate cortex is situated at the medial frontal lobe of the brain. It can monitor target orientation behavior [28], conflict and error monitoring [29, 30], behavioral decision-making and volitional control [31–36]. And it allocates attentional resources effectively in relevant brain regions according to the current task processing requirements [37]. Therefore, it is an advanced regulatory structure in the executive functional neural network [38]. In concordance with this, non-invasive imaging implies that the cingulum bundle is involved in executive control, as well as emotion, pain, and episodic memory. Clinical studies indicate that abnormalities in the cingulum may manifest as numerous conditions, including post-traumatic stress disorder schizophrenia, depression, obsessive-compulsive disorder, mild cognitive impairment, autism spectrum disorder, and Alzheimer’s disease [39]. Moreover, one study detected significant cortical atrophy in patients with AMD at long-term follow-up, with a decline in mean cortical volume across the whole occipital lobe. And what’s significant is this decline could be explained by cortical thinning of the lesion projection zone [40].

Resting-state fMRI and fractional ALFF scores were utilized by a former study to explore the neural mechanism of perceived stress in 234 healthy adolescents [41]. A positive correlation was found between perceived stress grades and the fractional ALFF measures at the left middle frontal gyrus, which remained after adjusting for the influence of positive and negative emotions [42].

Importantly, the left middle frontal gyrus can facilitate the connection between perceived stress and depression [43]. Furthermore, an activation likelihood estimation meta-analysis found a negative correlation between migraine severity and GMV in the left middle frontal gyrus and the bilateral inferior frontal gyri. Consistent with this, GMV reduction in the left middle frontal gyrus is also related to the estimated frequency of headache [44]. These findings together with the present results suggest that nAMD patients are liable to suffer from mental health disorders and headaches. Clinicians should be aware of this potential link when treating and managing patients with nAMD. Also, Charles Bonnet syndrome (CBS) is generally defined as the occurrence of recurrent complex visual hallucinations. It is a prevalent condition in patients with nAMD. Some researches indicated that the overall prevalence of CBS in patients with nAMD to be 15.8%—around one in six [45]. While it’s unfortunate that when measured in a population of patients with nAMD, awareness of CBS is limited and it may be difficult for clinicians to link CBS with it. Thus, according to clinical practice, we should understand the fact that CBS is relatively common in patients with nAMD and should consider providing help and relief for them.

### Summary

As investigated above, we have discovered that declined GMV values in certain areas in patients with nAMD indicated that they might suffer from mental sickness. Thus, unusual alternation in those areas can be symbolized as valuable clinical indices. However, our article also has some limitations. For example, we only took samples from one hospital and the number of samples was also small, which presented limitations of research scope and sample size. Also, while we have found loss of frontotemporal volume in the nAMD cases studied here which was associated with mildly raised HADS scores, the statistical approach used is suboptimal and liable to false positives. In the future, we will use more scientific methods to solve the problem.

## MATERIALS AND METHODS

This study recruited eighteen (9 men, 9 women) patients with nAMD from the Nanchang University’s First Affiliated Hospital. HADS scores were collected from those patients. They are composed of 14 items, including 7 items of self-rated anxiety scale and 7 items of self-rated depression scale. The inclusion criteria are as follows: (1) age ≥ 55 years old; (2) meeting the diagnostic criteria for nAMD. Patients meeting the following criteria were excluded: (1) history of ocular trauma or surgery; (2) previous treatment for nAMD such as intravitreal injection with anti-VEGF; (3) other retinal vascular diseases; (4) other ocular diseases decreasing the efficacy of injection; (5) history of myocardial infarction, stroke or other systemic diseases that are contraindications for injection.

**Figure 5:**
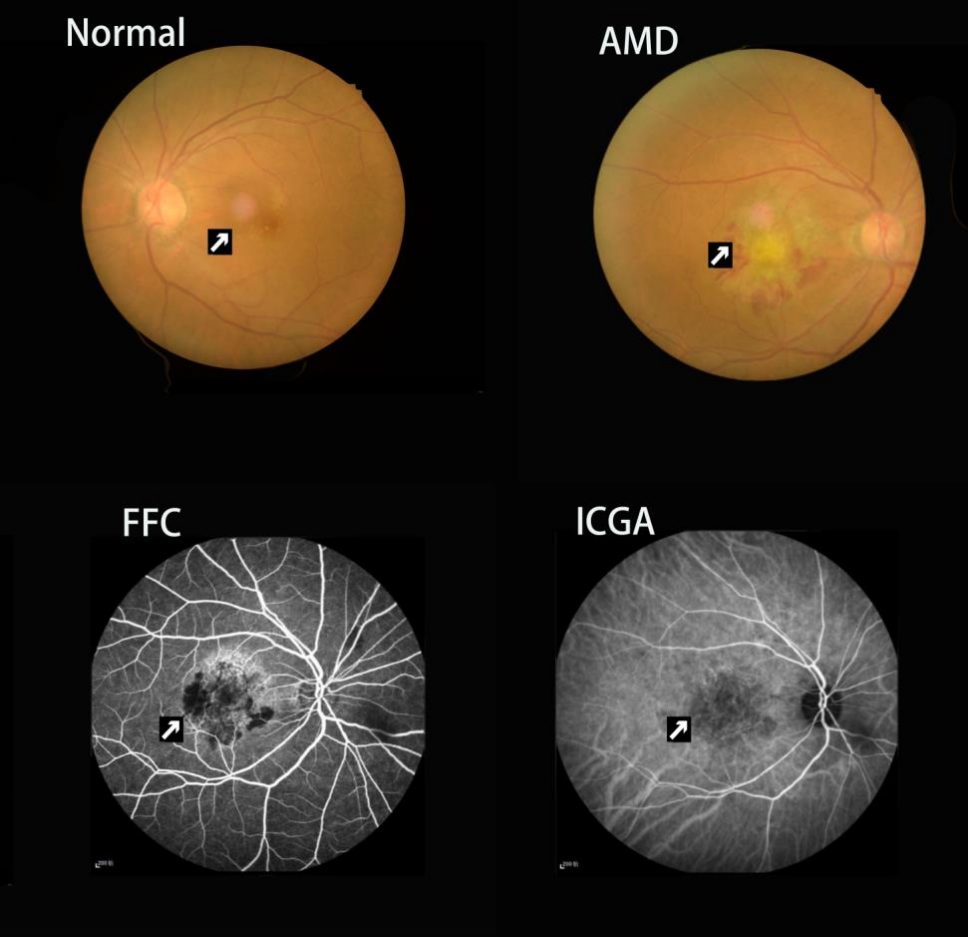
**Example of choroidal neovascularization and macular scar caused by neovascular age-related macular degeneration seen on fundus camera and fluorescence fundus angiography.** Abbreviations: FC, fundus camera; FFA, fluorescence fundus angiography; ICGA, indocyanine green angiography.

Eighteen age- and education-matched healthy control subjects (9 men, 9 women) were recruited based on the following criteria: (1) no ocular diseases (such as maculopathy, diabetic retinopathy, cataract or glaucoma); (2) corrected monocular visual acuities >1.0 decimal; (3) no indications for fMRI. The patients and their family members were familiarized with the research protocol and signed a declaration of informed consent before each procedure (Figure 5). The study was conducted in accordance with the tenets of the Declaration of Helsinki and was approved by the medical ethics council of the Nanchang University’s First Auxiliary Hospital.

### MRI parameters

To perform the MRI scanning, a three-Tesla scanner (Trio; Siemens, Munich, Germany) was utilized to record high-definition cross-sectional weighted T1 images. The whole-brain image was obtained with a rapid-gradient echo sequence in 176 1.0 mm slices with the following parameters: echo time=2.26 ms; replication time=1900 ms; flip angle=9°; visual field=215×230 mm; gap=0 mm. The whole scanning process was operated by the same neuroradiologist throughout.

### Image processing

MRIcro software (www.MRIcro.com) was used to eliminate fragmentary data. The configurable images were analyzed using statistical parametric mapping (SPM 8) (http://www.fil.ion.ucl.ac.uk) tools within MATLAB 7.9.0. Software (R2009b; The Mathworks, Inc., Natick, MA, USA). Using (VBM8) (http://dbm.neuro.uni-jena.de/vbm8/), the brain regions can be separated into white matter (WM), gray matter (GM), and cerebrospinal fluid (CSF) utilizing the default estimation selections (minimum deflection normalization; 60 mm interception for the calculation of Gaussian evenness of graphic intention; and the original European template for affine conversion of the International Consortium for Brain Mapping [ICBM]). The Diffeomorphic Anatomical Registration Through Exponentiated Lie (Dartel) algebra method in VBM8 was used for the normalization of the Montreal Neurological Institute (MNI) standard space. The Dartel method was used to generalize the GM and WM templates and normalize the GM and WM of each participant with these templates. A 6 mm full-width half-maximum Gaussian was used for smoothing. Standardized, adjusted and flattened graphs were subjected to group-standard analysis.

### Statistical analysis

After accounting for age and sex, we conducted a general linear model (GLM) analysis with the SPM8 toolbox to compare GM and WM between patients with nAMD and HCs. Gaussian random field (GRF) theory was used for large-scale comparison rectification (GRF calibrated, minimal z>2.3, voxel standard P<0.01, cluster standard P<0.05). To generate color-coded images, the voxels with statistical significance were superimposed on the fast acquisition gradient echo sequence (3DT1WI) of standardized three-dimensional images.

The receiver operating characteristic (ROC) curves of each brain region were compared to evaluate the average redistribution value. The objective of correlation analysis was to explore the relationship between the values in different regions (P < 0.05).

### Brain behavior

Based on the VBM results, REST software (version 1.8) was utilized to partition distinct brain regions into multiple regions of interest (ROIs). Within each ROI, the median gray matter volume (GMV) value of all voxels was calculated. In the nAMD group, correlation analyses were used to look for associations between the median GMV values and clinical symptoms.

### Clinical behavior

Clinical data including nAMD disease duration (months since the first symptom) and hospital anxiety depression scale (HADS) scores were collected. The SPSS 20.0 software was used to compare data using an independent samples t-test.

In all analyses, P values less than 0.05 were considered statistically significant.

### Ethical statement

All research methods were approved by the committee of the medical ethics of the First Affiliated Hospital of Nanchang University and were in accordance with the 1964 Helsinki declaration and its later amendments or comparable ethical standards. All subjects were explained the purpose, method, potential risks and signed an informed consent form.
