# Correction for: Decreased gray matter volume and increased white matter volume in patients with neovascular age-related macular degeneration: a voxel-based morphometry study

**DOI:** 10.18632/aging.203702

**Published:** 2021-11-14

**Authors:** Yan-Kun Shen, Qian-Min Ge, Yi-Cong Pan, Hui-Ye Shu, Li-Juan Zhang, Qiu-Yu Li, Rong-Bin Liang, Yi Shao, Yao Yu

**Affiliations:** 1 Department of Endocrinology and Ophthalmology, The First Affiliated Hospital of Nanchang University, Jiangxi Center of National Ocular Disease Clinical Research Center, Nanchan, Jiangxi, 330006, People’s Republic of China

**Keywords:** correction

Original article: Aging. 2021; 13:23182–23192.  . https://doi.org/10.18632/aging.203610

**This article has been corrected:** The authors asked to change the order of first and second authors:

Old authors list:

Yan-Kun Shen^1,*^, Qian-Min Ge^1,*^, Yi-Cong Pan^1,*^, Hui-Ye Shu^1^, Li-Juan Zhang^1^, Qiu-Yu Li^1^, Rong-Bin Liang^1^, Yi Shao^1^, Yao Yu^1,&^

New authors list:

Qian-Min Ge^1,*^, Yan-Kun Shen^1,*^, Yi-Cong Pan^1,*^, Hui-Ye Shu^1^, Li-Juan Zhang^1^, Qiu-Yu Li^1^, Rong-Bin Liang^1^, Yi Shao^1^, Yao Yu^1,&^

